# Efficiency of NHEJ-CRISPR/Cas9 and Cre-LoxP Engineered Recombinant Turkey Herpesvirus Expressing *Pasteurella multocida* OmpH Protein for Fowl Cholera Prevention in Ducks

**DOI:** 10.3390/vaccines11091498

**Published:** 2023-09-18

**Authors:** Nisachon Apinda, Yongxiu Yao, Yaoyao Zhang, Anucha Muenthaisong, Kanokwan Sangkakam, Boondarika Nambooppha, Amarin Rittipornlertrak, Pongpisid Koonyosying, Venugopal Nair, Nattawooti Sthitmatee

**Affiliations:** 1 Laboratory of Veterinary Vaccine and Biological Products, Faculty of Veterinary Medicine, Chiang Mai University, Chiang Mai 50200, Thailand; nisachon.a@cmu.ac.th (N.A.); anucharham@gmail.com (A.M.); kanokwansangkakam@gmail.com (K.S.); boondarika.n@cmu.ac.th (B.N.); amarin.r@cmu.ac.th (A.R.); pongpisid_koo@cmu.ac.th (P.K.); 2 The Pirbright Institute, Woking GU24 0NF, UK; yongxiu.yao@pirbright.ac.uk (Y.Y.); yaoyao.zhang@pirbright.ac.uk (Y.Z.); venugopal.nair@pirbright.ac.uk (V.N.); 3 Office of Research Administration, Chiang Mai University, Chiang Mai 50200, Thailand; 4 Jenner Institute, University of Oxford, Oxford OX1 2JD, UK; 5 Department of Biology, University of Oxford, Oxford OX1 3SZ, UK

**Keywords:** Cre-Lox, CRISPR-Cas9, HVT, fowl cholera, NHEJ, *Pasteurella multocida*, viral vector

## Abstract

Fowl cholera is caused by the bacterium *Pasteurella multocida*, a highly transmissible avian ailment with significant global implications, leading to substantial economic repercussions. The control of fowl cholera outbreaks primarily relies on vaccination using traditional vaccines that are still in use today despite their many limitations. In this research, we describe the development of a genetically engineered herpesvirus of turkeys (HVT) that carries the OmpH gene from *P. multocida* integrated into UL 45/46 intergenic region using CRISPR/Cas9-NHEJ and Cre-Lox system editing. The integration and expression of the foreign cassettes were confirmed using polymerase chain reaction (PCR), indirect immunofluorescence assays, and Western blot assays. The novel recombinant virus (rHVT-OmpH) demonstrated stable integration of the OmpH gene even after 15 consecutive in vitro passages, along with similar in vitro growth kinetics as the parent HVT virus. The protective efficacy of the rHVT-OmpH vaccine was evaluated in vaccinated ducks by examining the levels of *P. multocida* OmpH-specific antibodies in serum samples using ELISA. Groups of ducks that received the rHVT-OmpH vaccine or the rOmpH protein with Montanide™ (SEPPIC, Paris, France) adjuvant exhibited high levels of antibodies, in contrast to the negative control groups that received the parental HVT or PBS. The recombinant rHVT-OmpH vaccine also provided complete protection against exposure to virulent *P. multocida* X-73 seven days post-vaccination. This outcome not only demonstrates that the HVT vector possesses many characteristics of an ideal recombinant viral vaccine vector for protecting non-chicken hosts, such as ducks, but also represents significant research progress in identifying a modern, effective vaccine candidate for combatting ancient infectious diseases.

## 1. Introduction

*Pasteurella multocida* (*P. multocida*) is the causative agent of fowl cholera, a highly contagious and fatal disease that affects a wide range of avian species, including both wild birds and domestic poultry such as chickens, ducks, and waterfowl [1]. In the duck industry, this disease is of global concern due to its high contagiousness. Notably, *P. multocida* carriers are prevalent in healthy duck flocks, with rates as high as 63%, and it can lead to a high mortality rate of up to 50%, resulting in significant ecological and economic challenges for the duck industry [2]. Infections are primarily associated with capsular type A strains of *P. multocida*, although occasional involvement of other capsular types has been reported [3]. Vaccination remains a vital and effective strategy for disease prevention, playing a crucial role in reducing economic losses in the poultry industry. However, currently available vaccines against fowl cholera, including bacterins, live attenuated vaccines, and traditional vaccines, have inherent limitations such as limited duration of immunity, safety concerns, and reduced efficacy [4]. Consequently, there is a pressing need to explore innovative vaccine design strategies capable of eliciting enhanced cross-protective immune responses, which hold the promise of overcoming these limitations and potentially revolutionizing fowl cholera prevention and control [5].

Outer membrane protein H (OmpH) plays a crucial role in *P. multocida* due to its function as an adhesion protein, facilitating the binding of bacteria to host cells during the early stages of infection. Furthermore, OmpH stands out as a significant point of interest in the host’s immune defense, underscoring its importance in the context of pathogenesis and potential vaccine development. Numerous previous studies have shown that the use of recombinant OmpH (rOmpH) effectively stimulates robust protection in immunized chickens and ducks, resulting in a substantial induction of antibody-mediated protection [6,7,8,9]. Another major immunogenic antigen, OmpA, acts as a cross-protective antigen and serves as an outer membrane protein of *P. multocida*. It has demonstrated potential in reducing mortality in experimental animals when targeted using monoclonal antibodies. However, prior studies have also highlighted that, despite OmpA’s capacity to induce a high-level immune response, some research findings have not consistently demonstrated ideal protective effects. Consequently, the immunological impact of the OmpA protein from *P. multocida* remains inconclusive [10,11].

Viral vector vaccines offer a fusion of the safety advantages inherent in inactivated vaccines and the immunological benefits and cost-effectiveness associated with live vaccines, establishing them as a well-established choice in veterinary medicine [12,13]. Nearly four decades ago, the development of viral vectors using the vaccinia virus commenced with the aim of creating vaccines [14]. In recent years, substantial progress has been made in genetically modifying the herpesvirus of turkey (HVT) to serve as a vaccine vector, effectively targeting a wide range of poultry diseases [15,16,17]. As a naturally occurring apathogenic virus in turkeys with a long history of safe use in chickens, HVT is widely recognized as a highly suitable candidate for a vaccine that stimulates both humoral and cellular immunity, providing lifelong protection even in the presence of maternally derived antibodies [18]. Operating as a viral vector, it possesses the ability to incorporate foreign gene additions at numerous sites. Because of these advantages, there are many commercially available HVT-vectored vaccines with several stable genomic loci for gene insertion in the HVT genome, including US2, US10, and intergenic regions of UL45/UL46 and HVT065/HVT06 [18,19]. While all of these recombinant HVT vectored vaccines express protective antigens of viral origin such as Newcastle disease virus (NDV), infectious laryngotracheitis virus (ILTV), infectious bursal disease virus (IBDV), and avian influenza virus, it will be interesting to explore whether HVT can also express protective bacterial antigens to induce immunity against numerous bacterial diseases [16,17,18,19,20].

In this study, we harnessed the properties of the HVT UL45/UL46 intergenic region to create a potential vector capable of expressing the highly immunogenic OmpH antigen of *P. multocida*, utilizing the NHEJ-dependent CRISPR/Cas and Cre-Lox gene editing system. Subsequently, we conducted an assessment of the recombinant HVT-OmpH virus for its in vitro replication, OmpH expression, stability, safety profile, and protective efficacy against fowl cholera in ducks.

## 2. Materials and Methods

### 2.1. Virus Strain and Cell Culture

Primary chicken embryo fibroblasts (CEF) cells were maintained in M199 medium (Thermo Fisher Scientific, Waltham, MA, USA) supplemented with 5% fetal bovine serum (FBS, Sigma-Aldrich, Darmstadt, Germany), 100 units/mL of penicillin and streptomycin (Thermo Fisher Scientific), 0.25 µg/mL Fungizone (Sigma, Boston, MA, USA), and 10% tryptose phosphate broth (Sigma) and maintained at 37 °C with 5% CO_2_. The attenuated HVT Fc126 strain, acquired from the Pirbright Institute, Pirbright, UK, was used as the HVT wild type in this study.

### 2.2. CRISPR/Cas9-Mediated HVT-OmpH Cloning

The oligos of the gRNA targeting the HVT UL45/46 intergenic region and the sg-A sequence were synthesized and cloned into pX459-V2 (Addgene, UK) following the method stated previously [20]. The donor plasmid containing the removable green fluorescent reporter gene (GFP) and OmpH-V5 gene expression cassettes was designed and constructed previously [21]. The steps for transfection and HVT infection to create recombinant HVT were conducted according to the previously provided description [20]. After 72 h of infection, the virus was harvested and subjected to plaque purification using fluorescence-activated cell sorting (FACS) for a single green infected cell. The gRNA primers are listed in Table 1.

### 2.3. The Removal of the GFP Cassette from HVT-GFP-OmpH-V5 Was Achieved Using the Cre-Lox System

For the excision of GFP, 2 µg of Cre recombinase plasmid (pcDNA3-Cre) was transfected into CEF cell in the 24-well plate. 24 h post-transfection, the cells were infected with 100 pfu of HVT-GFP-OmpH-v5. Three days later, GFP-negative plaques were selected and employed to infect fresh CEF cells in 6-well plates for subsequent purification.

### 2.4. Analysis of the Properties of HVT-OmpH-V5 Recombinant Viruses

CEF cells were pre-seeded into 6-well plates and subsequently inoculated with purified HVT-GFP-OmpH-V5. After 72 h of infection, the cells were harvested and treated with squishing buffer at 65 °C for 30 min, with the reaction stopped by heating at 95 °C for 5 min, as previously described [20]. To verify the proper foreign gene insertion in recombinant HVT-GFP-OmpH-V5, PCR was conducted targeting the junction regions by employing specific primers UL45-F and UL46-R. Additionally, junction PCR identification of recombinant HVT-OmpH-V5 viruses was carried out using primer pairs UL45-F and OmpH-5R, as well as OmpH-3F and UL46-R. A comprehensive list of all primers used can be found in Table 1.

### 2.5. Western Blot Analysis

Western blotting was employed to demonstrate the expression of OmpH proteins in CEF cells infected with recombinant HVT. Initially, infected cells were boiled using TruPAGE™ Precast Gels supplemented with LDS sample buffer (Sigma, Boston, MA, USA) for 10 min, and the separated SDS-PAGE bands were then transferred onto PVDF membranes. Subsequently, immunoblots were blocked with 5% skimmed milk for 1 hour at room temperature and washed three times with Tris-buffered saline (TBST). Following this, the membranes were incubated with anti-OmpH antibodies (1:1000) overnight at 4 °C on a rocker. Afterward, the blots were once again washed with TBST and incubated with the secondary antibody IRDye^®^680RD goat anti-mouse IgG (LI-COR, USA) at a dilution of 1:5000 at 37 °C for 2 h. Images were visualized and analyzed using the Odyssey Clx imaging system (LI-COR, Lincoln, NE, USA).

### 2.6. Indirect Immunofluorescence Analysis (IFA)

Immunofluorescence assays with immunocytochemistry were employed to assess the expression of the V5-tag in CEF cells infected with recombinant viruses. CEF cells were cultured in 24-well plates and infected with both the parental virus and each recombinant virus at an MOI of 0.01 for 48 h prior to harvesting. The V5-tag expression was analyzed using a monoclonal mouse anti-V5 antibody (Bio-Rad, Hercules, CA, USA) after fixing the cells with ice-cold acetone:methanol (1/1) for 10 min. As a positive control for detecting HVT infection, MAb anti-HVT-gB L78 was used. We utilized a blend of secondary antibodies, which included goat anti-chicken IgG tagged with Alexa Fluor 488, along with goat anti-mouse IgG tagged with Alexa Fluor 568 (Invitrogen, Waltham, MA, USA), for the identification of targeted fluorescence. Images of cells displaying positive staining were recorded using an IncuCyte analyzer (Sartorius, Germany), covering 36 discrete regions within each well for every sample.

### 2.7. Assessment of Gene Insert Stability in Recombinant Viruses

The recombinant viruses underwent 15 consecutive passages in triplicate in 6-well plates with CEF cells. Immunofluorescence assays (IFA) were performed to assess the V5 expression at each consecutive 5th passage. Additionally, in order to confirm the soundness of the OmpH gene insertion, DNA was extracted from each 5th passage, and PCR was conducted using primer pairs UL45-F and UL46-R, targeting the junction of the inserted OmpH-V5 cassette.

### 2.8. In Vitro Growth Kinetics

CEF cells were infected with both HVT and recombinant HVT-OmpH-V5 at a multiplicity of infection (MOI) of 0.01, with triplicate wells in 6-well plates. Cells were collected at different time intervals: 0, 12, 24, 48, 72, 96, and 120 h post-infection. The gathered viral samples were preserved at −80 °C until subsequent analysis. DNA was extracted using the DNeasy 96 Blood and Tissue kit (Qiagen, Hilden, Germany), and the in vitro growth kinetics of the viruses were assessed using real-time qPCR, as per previously established methods [20].

### 2.9. Immunogenicity and Protection of rHVT-OmpH-V5 in Ducks

A total of 40 Khaki Campbell ducks (3 weeks old), seronegative for fowl cholera, were obtained from a commercial hatchery and were randomly divided into ten ducks of four groups (Table 2). Ducks in groups A and B were inoculated intramuscularly with two doses (0.5 mL/dose) at 4-week intervals, each at a dose of 3000 pfu/mL with rHVT-OmpH-V5 and HVT vaccine, respectively. Ducks in groups C and D were inoculated intramuscularly at the same time with 100 mg of rOmpH mixed with Montanide^TM^ (SEPPIC, Paris, France) and 0.5 mL of PBS as positive and negative controls, respectively. Throughout the post-immunization period, serum samples from all ducks were collected every two weeks for the assessment of *P. multocida* serological responses using the ELISA test, as per the methodology described in a previous study [6]. At 4 weeks post-final immunization, all the groups were inoculated intramuscularly challenged with virulent *P. multocida* strain X-73 (ATCC#11039) at a dose of 3.5 × 10^6^ cfu/mL and daily observed for 7 days to analyze the morbidity and mortality. All ducks were humanely sacrificed on day 7 post-challenge. Then, a post-mortem examination was conducted, and tissue samples were aseptically collected to avoid cross-contamination for *P. multocida* bacterial isolation. The sample size was calculated using the G*Power program (Version 3.1.9.2) with a desired statistical power set at 0.8 and a significance level (α) of 0.05.

The utilization of laboratory animals in this study was conducted in compliance with the regulations set forth by the animal welfare committee of the Faculty of Veterinary Medicine, Chiang Mai University. All animal experiments adhered to the principles outlined in the Guide for the Care and Use of Agricultural Animals in Research and Teaching (the Ag Guide, FASS 2010). Experiments involving virulent HVT were conducted under Biosafety Level 2+ containment.

### 2.10. Statistical Analysis

Statistical analysis was performed using GraphPad Prism 6 software (GraphPad Software, La Jolla, CA, USA). One-way ANOVA was employed to evaluate distinctions among different groups. Furthermore, the Mantel–Cox test was utilized for comparing survival outcomes across groups. Statistical significance was established at a *p*-value of less than 0.05.

## 3. Results

### 3.1. Rapid Generation of Recombinant HVT-OmpH-V5 Virus Based on CRISPR/Cas9 Mediated Gene Editing

**Figure 1:**
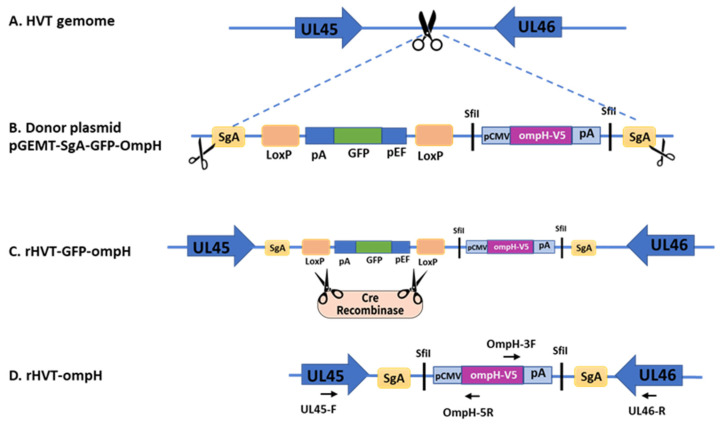
Schematic diagram of recombinant HVT-OmpH construction. (**A**) The intergenic region between UL45 and UL46 of the wild-type (wt) Herpesvirus of turkey (HVT) was targeted by the single guide (sg)RNA, inducing double-stranded breaks (DSBs) in the viral genome. (**B**) Donor plasmid or pGEMT-SgA-GFP-OmpH were flanked with sg-A targeting both sites to release the belt cassette and integrate into the genome of HVT using the NHEJ-CRISPR/Cas9 strategy. The donor plasmid encompasses both the GFP expression cassette, bordered by LoxP sites, and the OmpH-V5 expression cassette. The presence of the sgRNA of 45/46 and sgA targeting sites in the donor plasmid is indicated by the scissor symbol (blacks). (**C**) The first-generation recombinant HVT is called rHVT-GFP-OmpH. Next, the GFP reporter expression cassette was removed from rHVT-GFP-OmpH via the utilization of the Cre-loxP system. (**D**) The newly generated recombinant HVT was designated as rHVT-OmpH. The arrows indicate the specific primers employed for the verification of the recombinant virus.

Earlier research has illustrated that the intergenic region spanning HVT UL45 and UL46 presents a conducive locus for integrating foreign genes without causing any adverse effects on viral replication [20,22]. Based on this evidence, we selected this specific site as the target locus for creating the recombinant HVT-OmpH-V5 (Figure 1A).

The individual single guide RNAs (sgRNAs) targeting the intergenic region UL45/46 and SgA targeting the donor plasmid to release the insert fragment were cloned into pX459-v2, which contains Cas9 gene from *S. pyogenes* for the gRNA cloning vector. The donor plasmid, depicted in Figure 1B, was adapted from a previous study and includes the GFP reporter gene and OmpH-V5 cassettes flanked by the SgA target sites [21]. The simultaneous cleavage of both the donor plasmid DNA and the viral genome by Cas9 leads to the insertion of the GFP-OmpH-V5 cassette into the UL45/46 region. Additionally, single-cell fluorescence-activated cell sorting (FACS) was employed to aid in the purification of plaques during the experimental process. By utilizing CRISPR/Cas9-mediated recombination, individual cells exhibiting positive GFP signals were sorted into a 96-well plate that had been pre-seeded with chicken embryo fibroblast (CEF) cells. The GFP-positive progeny virus was subsequently purified using three rounds of plaque purification. The purified recombinant HVT-OmpH of the first generation was designated as rHVT-GFP-OmpH (Figure 1C). After that, the GFP expression cassette was removed using Cre treatment as described previously [21]. Finally, the purified new recombinant HVT was named rHVT-OmpH after being identified by PCR using outside specific insertion sites primers UL45-F and UL46-R (Figure 1D).

**Figure 2:**
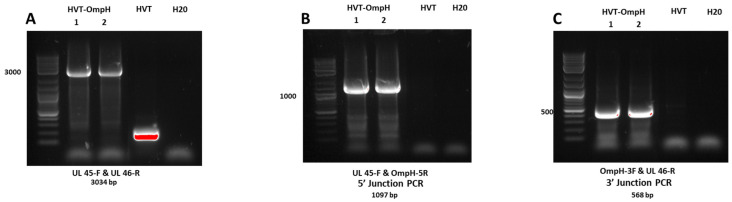
Identification of recombinant HVT-OmpH by PCR. (**A**) PCR analysis was conducted for the purification of the inserted foreign gene cassettes, utilizing primers positioned at the flanking regions of the insertion sites. The higher bands (about 3000 bp) demonstrate the presence of foreign gene cassettes in recombinant viruses, whereas the lower bands (red) reveal the PCR product without foreign gene insertion. (**B**,**C**) The correct insertion and integration of the OmpH genes in the recombinant HVT virus were verified using 5′ and 3′ junction PCR, respectively, utilizing the primers illustrated in Figure 1D. The presence of positive bands indicated the successful insertion in the recombinant viruses, with the corresponding molecular sizes indicated on the left.

As depicted in Figure 2A, the PCR analysis did not detect the wild-type HVT-specific band in the sorted population cells infected with purified rHVT-OmpH, in contrast to HVT wild-type-infected CEF cells. This outcome indicates that the CRISPR/Cas9 system is a straightforward and remarkably effective approach for integrating foreign genes into the HVT genome. Moreover, the insertion at the correct locus was verified by PCR using junction primers. The results showed both 5′and 3′ junction PCR for OmpH-V5 insertion were amplified from all recombinant viruses, confirming the correct integration location and orientation, whereas no PCR product was amplified for wild-type HVT and negative H_2_0 at any junction (Figure 2B,C). All specific primers are shown in Table 1.

### 3.2. Expression of the OmpH-V5 in CEF Cells Infected with the Recombinant HVT-OmpH Viruses

**Figure 3:**
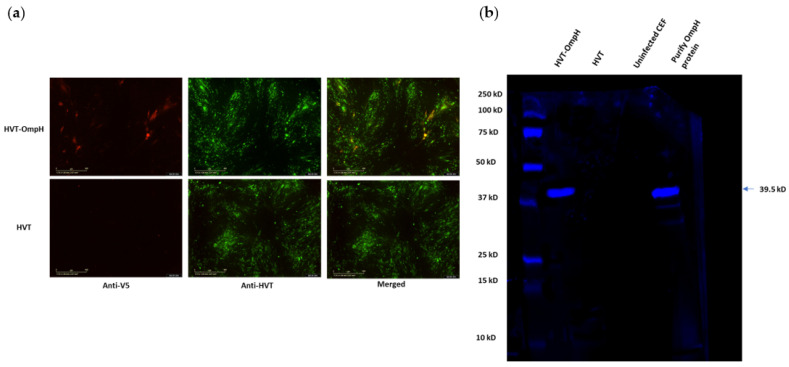
The expression of the OmpH-V5 in CEF cells infected with rHVT-OmpH. (**a**) Detection of V5 expressions by immunofluorescence. Fluorescence microscopy revealed positive red (Anti-V5) and green (Anti-HVT) fluorescence signals in recombinant virus-infected CEFs, while HVT-infected cells did not exhibit such signals. Upon merging the two fluorescent images, co-localization of the green and red fluorescence signals was observed in the same CEF cells. (**b**) Verification of OmpH protein expression using immunofluorescence analysis. CEF cells infected with HVT-infected CEF cells and uninfected were used as negative controls, whereas the purified OmpH protein was used as positive control. The Western blot was incubated with anti-OmpH antibodies, probed with the secondary antibody IRDye^®^680RD goat anti-mouse IgG, and visualized using the Odyssey Clx imaging system (LI-COR, USA). The arrowhead pointed to the positive protein. The molecular weight of marker proteins is indicated on the left.

The expression of V5-tagged OmpH in CEF cells infected with rHVT-OmpH was examined using immunofluorescence assay (IFA). HVT-infected cells were used as a negative control. As shown in Figure 3a, both positive red (Anti-V5) and green (Anti-HVT) fluorescence signals were observed using fluorescence microscopy in rHVT-OmpH-infected CEF, but HVT-infected cells only showed green staining. Upon combining both fluorescent images, the green and red fluorescence signals were observed to co-localize in the same CEF cells.

Moreover, the expression of OmpH protein in CEF cells infected with recombinant HVT-OmpH was also investigated using Western blot. As expected, the band corresponding to the molecular size of OmpH (39.5 kDa) was visible in the lysate of the rHVT-OmpH-infected CEF cells (Figure 3b). Conversely, OmpH was not detected in lysate CEF cells that had been infected with HVT or un-infected. These results indicate that the OmpH proteins were expressed in the recombinant HVT-OmpH-infected CEF cells.

### 3.3. Stability of Recombinant HVT-OmpH

**Figure 4:**
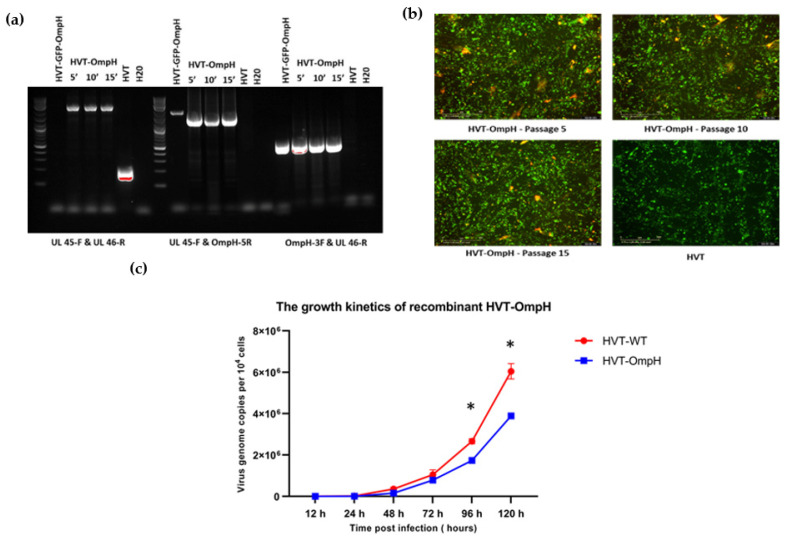
The stability and replication kinetics detection of rHVT-OmpH. (**a**) PCR with the specific primer pairs between the insertion site and junction PCR was performed on the 5th, 10th, and 15th generations of recombinant virus. HVT and H_2_O were used as templates for negative control. (**b**) The expression of V5 protein was identified by IFA on the 5th, 10th, and 15th continual passages. Merged images of double-stained V5 (red) and HVT-GB (green) staining were taken using the IncuCyte. (**c**) One-step growth curve of rHVT-OmpH. The harvested infected cells were collected at 12, 24, 48, 72, 96, and 120 h post-infection (hpi) and quantified using the plaque assay. The results are presented as the mean ± SD. Significance is denoted using an asterisk (*) for values of *p* < 0.05.

The recombinant HVT-OmpH was propagated using 15 consecutive passages in CEF cells. After every set of 5 passages, samples were collected, and the genetic stability of rHVT-OmpH during its expansion in CEF cells was verified using PCR and IFA analysis. PCR analysis confirmed the stable insertion and consistent maintenance of the OmpH gene within the vector across all 15 passages (Figure 4a). Furthermore, immunofluorescence assays demonstrated a consistent expression of the V5-tag and MAb anti-HVT-gB L78, which served as the control for HVT infection, with double-stained cells detected in all passages (Figure 4b).

In terms of growth kinetics, rHVT-OmpH demonstrated comparable characteristics to the HVT vector, as evident from genome copy measurements at various time points post-infection (Figure 4c). Both rHVT-OmpH and the original HVT strain exhibited slight growth rate enhancements from 24 h to 72 h, followed by a more noticeable increase from 72 h to 120 h. However, it’s important to highlight that the growth rate of the original HVT consistently maintained a slight edge over that of rHVT-OmpH, with a notable difference emerging at 96 and 120 h after infection. This observation suggests that the presence of the OmpH cassette encoded in HVT might contribute to the deceleration of the virus replication cycle.

### 3.4. Induction of Antibody Response in Recombinant HVT-OmpH Vaccinated Ducks

**Figure 5:**
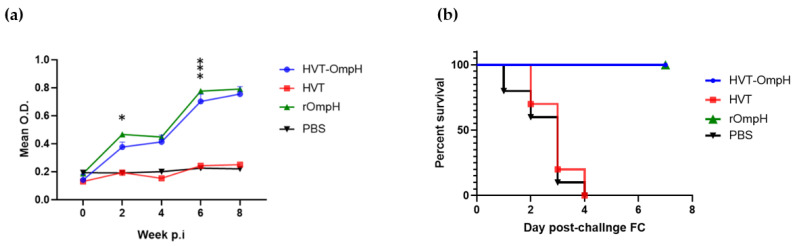
Assessment of the potential rHVT-OmpH vaccine against fowl cholera in ducks. (**a**) ELISA analysis was conducted at 450 nm to compare serum IgG levels among vaccinated ducks with rHVT-OmpH, HVT, rOmpH (positive control), and PBS (negative control group). Each bar represents the mean serum activity of individual ducks (n = 10). (**b**) Survival rates of vaccinated ducks in each group were evaluated following the challenge with *P. multocida* strain X-73. Significance is indicated using asterisks (* for *p* < 0.05, *** for *p* < 0.001).

Levels of antibodies in ducks that received immunization with rHVT-OmpH were evaluated using an indirect ELISA, utilizing serum samples collected every two weeks after vaccination. As depicted in Figure 5a, the average antibody levels in ducks belonging to the rHVT-OmpH group surpassed the cut-off threshold two weeks after vaccination and exhibited a significant rise at 6 and 8 weeks after vaccination. Conversely, the mean antibody values of the PBS control and HVT groups remained below the cut-off value throughout the course of the experiment.

### 3.5. Evaluation of Protection Post-Challenge with P. multocida Strain X-73

As shown in Figure 5b, ducks immunized with rHVT-OmpH via the IM route showed numerically greater survival rates than the challenge parent HVT-immunized and control group. Furthermore, ducks immunized with rHVT-OmpH exhibited no clinical signs and showed no apparent lesions in the liver, spleen, or other organs throughout the one-week observation period following the challenge with *P. multocida* strain X-73. In stark contrast, all ducks in the challenge control and HVT groups displayed severe clinical signs, such as depression, lethargy, and anorexia, within 1–2 days post-challenge. Tragically, all these ducks either succumbed to the infection or had to be euthanized within 4 days. In our study, we conducted a log-rank test (Mantel-Cox) to rigorously evaluate the survival data obtained from the experimental and control groups. The log-rank test revealed a highly significant difference in survival between these groups (χ^2^ = 42.34, df = 3, *p* < 0.0001), clearly demonstrating the substantial impact of the vaccine on survival outcomes. This statistical analysis underscores the remarkable effectiveness of the rHVT-OmpH viral vector vaccine in enhancing the survival rates of immunized ducks when compared to the control group. Interestingly, the protection observed with rHVT-ompH was similar to that induced by the immunization of ducks with the r-OmpH antigen alone. These results demonstrated that rHVT-OmpH induced 100% protection against the lethal *P. multocida* strain X-73 challenge in ducks.

**Figure 6:**
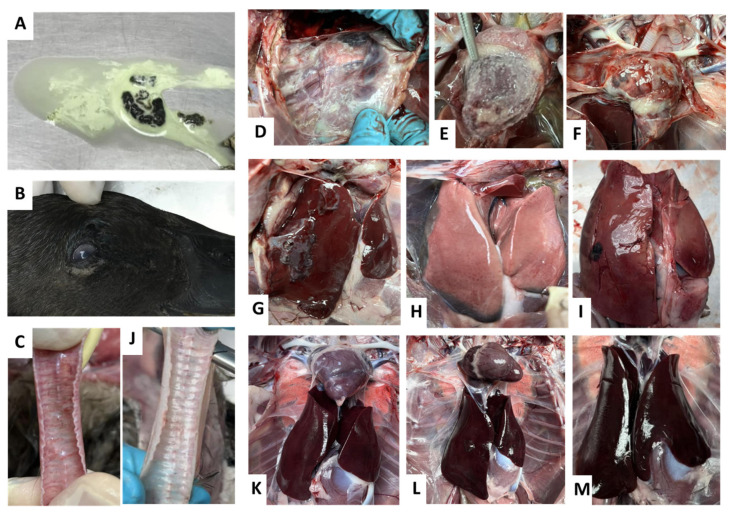
Clinical and Gross lesions in ducks experimentally infected with *P. multocida* X-73. (**A**) Diarrhea (**B**) Blepharoconjunctivitis, hypopyon (**C**) tracheitis (**D**–**F**) severe lesions of septicemia and exudative fibrinous polyserositis on (**D**) airsacculitis (**E**) pericarditis (**F**) myocarditis (**G**) perihepatitis (**H**,**I**) hepatitis with petechial and focal hemorrhages were found in ducks HVT vaccinated and control group. At the same time, no clinical signs and pathognomonic of disease were found in ducks rHVT-OmpH and rOmpH vaccinated groups as (**J**) normal trachea (**K**) Serosal membrane (**L**) normal airsac and heart (**M**) normal liver.

On post-mortem examination, all study groups were assessed for the presence of gross lesions. Severe lesions in the lungs, airsacs, and spleens were observed in all ducks belonging to the control and parental HVT groups. Almost all ducks showed gross pathological lesions, had exudative fibrinous polyserositis, remnants of fibrin and fibrous strands on the airsacs and pericardium, and some ducks had necrotic spots on the spleen and liver. Moreover, *P. multocida* was isolated from all dead ducks in both groups. Conversely, the rHVT-OmpH and rOmpH protein-inoculated groups exhibited no lesions in the lungs and thoracic airsacs. The average gross lesion scores of lungs and thoracic airsacs in the rHVT-OmpH and rOmpH protein-inoculated groups were significantly lower compared to the other groups. Additionally, no evident histopathological lesions were observed in the thoracic airsacs and spleens of the rHVT-OmpH group (Figure 6).

Moreover, we have also examined the replication of HVT by PCR in ducks. Employing specific primers targeting the glycoprotein A (gA) gene, we successfully amplified a 388 bp DNA fragment from the HVT genome, consistent with findings from a prior study [23]. Interestingly, the inability to detect specific sizes of PCR product from the tissue sample (spleen) of the vaccinated groups would mean that the antibody detection and protection are simply from the expression of the OmpH protein without HVT replication in ducks.

## 4. Discussion

*P. multocida*-induced fowl cholera presents a significant global challenge due to its severity and high contagiousness in various avian species, including ducks. In Thailand, the presence of free-ranging or free-grazing ducks increases the risk of exposure to *P. multocida* from carrier migratory fowl. Vaccination plays a crucial role in disease prevention and the reduction in economic losses in the poultry industry [24]. However, developing an effective vaccine for fowl cholera has been a longstanding challenge. An ideal vaccine should not only be safe but also provide long-lasting protection, ultimately leading to the elimination of the disease challenge. Currently, the inactivated fowl cholera (FC) vaccine in the form of a broth bacterin is widely used in Thailand following the Department of Livestock Development recommendation. However, inactivated vaccines exhibit limited immunogenicity, requiring supplementary booster doses and the incorporation of adjuvants such as oils, saponins, and aluminum hydroxide to confer extended immunity [25,26]. Furthermore, immune responses triggered by inactivated vaccines predominantly center on humoral immunity, which has a gradual onset, rendering them unsuitable for a DIVA (Differentiating Infected from Vaccinated Animals) approach [24,27].

Various viral vectors, including fowl pox virus (FPV), turkey herpesvirus (HVT), adenovirus, ILT, and MDV, have been well-established and employed in the development of poultry viral vaccines [24]. In recent times, HVT-based vectors have gained increasing attention in the realm of vector vaccines due to their superior efficacy. Recombinant HVT vaccines have demonstrated exceptional phenotypic stability, maintaining their avirulent nature and exhibiting rare horizontal transmission [28]. Moreover, these vaccines exhibit efficacy even in the presence of existing maternal antibodies within young animals [29,30]. Their capacity to endure in the face of elevated maternal antibody levels has facilitated their successful deployment as viral vector vaccines. Via the expression of NDV F and HN proteins, these vaccines efficiently decrease viral shedding in vaccinated birds, eliciting enduring cellular and humoral immunity with just a single-dose administration [31].

Previous studies on rHVT vaccines for ducks have primarily focused on Perkin ducks (*Anas platyrhynchos domesticus*) [32], mallard ducks (*A. platyrhynchos*) [33], and Muscovy ducks (*Cairina moschata*) [34]. However, there has been limited research on Khaki Campbell ducks. In a previous study involving Mulard ducks, it was observed that the rHVT-H5 vaccine elicited a modest antibody response to its specific antigen and undetectable levels for other tested viruses. Despite this, the vaccine proved effective and provided protection against challenging HPAI H5N1 clade 2.2.1 viruses. Additionally, rHVT-HA triggered both cellular and humoral immune responses, including cell-mediated immunity in ducks [35]. Out of the various tissues examined, the spleen proved to be the most suitable sample for HVT detection. Among the various tissues examined, the spleen was found to be the most suitable sample for detecting HVT. However, the amount of detected rHVT-AI within the examined organs was relatively modest [23]. This finding is consistent with our result, as we were unable to detect the specific HVT gene in the spleen of vaccinated ducks. These data are interesting as they demonstrate that HVT cannot replicate in Khaki Campbell ducks.

In this study, we generated rHVT-OmpH using the NHEJ-CRISPR/Cas9 and Cre-Lox system. Effectively, the OmpH expression cassettes were integrated into the UL45/UL46 region and consistently maintained within the recombinant viruses for up to 15 passages. These findings align with prior research that has similarly established UL45/UL46 as suitable sites for foreign gene integration [20,22,36]. Moreover, the possible application of multivalent rHVT vaccine in different species of poultry for the control of fowl cholera was proved using antibody response and protection efficacy against *P. multocida* in ducks. Here, we observed a significant decline in maternal immunity on or before 21 days of age. When making vaccination decisions against viral or bacterial infections in ducks, it is crucial to consider the waning of maternal immunity before administering vaccines. Interference between maternal immunity and vaccine-induced protection has been documented in poultry, underscoring the importance of proper timing for vaccination [34]. The efficacy evaluation showed that double inoculation with 3000 pfu/mL of rHVT-OmpH after 3 weeks of age could provide a high level of OmpH-specific antibodies until 8 weeks post-immunization, resulting in full protection without pathognomonic lesions in ducks challenged with *P. multocida* X-73. Previously, the rate of rHVT-AI vaccine vector replication was detected in several waterfowl species at 1 day old. Their study revealed varying degrees of persistent infection by the rHVT-AI vaccine in different waterfowl species. Throughout the tested post-vaccination period of 5 weeks, no detectable humoral immune response was observed using the HI test or ELISA. This suggests that cellular immunity might play a crucial role in providing protection [33]. When comparing recombinant HVT vector vaccines with inactivated vaccines, the rHVT-ND vaccine demonstrated superior protective immunity. This enhanced protection may be attributed to a robust Th1-mediated immune response, coupled with a humoral response, which proves more effective than the Th2-oriented response elicited by conventional vaccine-based vaccination programs. Both the recombinant HVT and inactivated vaccines demonstrated the capacity to confer protection even in the absence of a noticeable humoral immune response [37].

Using the rOmpH antigen alone simplifies vaccine production and reduces safety concerns, but it may result in a less robust and shorter-lived immune response. In contrast, the rHVT-OmpH viral vector vaccine offers advantages, including an enhanced immune response, longer duration of immunity, and multivalent potential. However, it does come with safety considerations. Our study has demonstrated the safety and efficacy of rHVT-OmpH in experimental ducks. The choice between these approaches depends on specific vaccine goals and safety considerations.

## 5. Conclusions

In conclusion, our study has demonstrated that the CRISPR/Cas9 system represents an innovative, uncomplicated, adaptable, and robust technology for targeted engineering of HVT viral vectors for vaccine development in other species of avian. Given the established enduring presence of OmpH-specific antibodies in immunized birds, we anticipate that the protection provided by rHVT-OmpH will extend beyond the initial 2 months post-vaccination. Nevertheless, future studies are warranted to allow for a more focused investigation into the specific antigenic properties of *P. multocida*, with the potential for developing multivalent vaccines. Our results open a new avenue in the development of recombinant vaccines using HVT as a vector for other foreign immunogenic gene pathogens that could be a future alternative to rHVT-based vectors in ducks.

## Figures and Tables

**Table 1:** The list of primers applied for the construction of gRNA, the donor plasmid, and the confirmation of insertion in the recombinant HVT * OmpH ** virus.

Primer	Sequences
sgRNA-UL45_46-F	CACCGAAAACACAGTAACCGTTAG
sgRNA-UL45_46-R	AAACCTAACGGTTACTGTGTTTTC
sg-A-gRNA-F	CACCGAGATCGAGTGCCGCATCAC
sg-A-gRNA-R	AAACGTGATGCGGCACTCGATCTC
UL45-F	GATGCCCGCGTGTATCTTCA
UL46-R	ACGTAGGCTGAAAGTGTCCAG
OmpH-3F	ACGTGCTCTTGAAGTGGGTT
OmpH-5R	GCGAAACCCGCATAAAGACG

* Genbank accession number: AF291866.1. ** Genbank accession number: U50907.1.

**Table 2:** Type of vaccine immunization and challenge exposure in duck experiment.

Group	Vaccination Formulation	Challenge Exposure (IM) Duck/Group
		*P. multocida* X-73 (3.5 × 10^3^ CFU/mL)
A	rHVT-Omp of 3000 pfu/mL ^a^	10
B	HVT vaccine ^b^	10
C	rOmpH 100 µg/ml	10
D	Phosphate-buffered saline (PBS)	10
Total		40

^a^ A recommended dose for the HVT vaccine. ^b^ A recommended dose of the commercial HVT live vaccine.

## Data Availability

All data in this study have been included in the manuscript.
